# Water, land, fire, and forest: Multi‐scale determinants of rainforests in the Australian monsoon tropics

**DOI:** 10.1002/ece3.2734

**Published:** 2017-02-09

**Authors:** Stefania Ondei, Lynda D. Prior, Grant J. Williamson, Tom Vigilante, David M. J. S. Bowman

**Affiliations:** ^1^ School of Biological SciencesUniversity of TasmaniaSandy BayTas.Australia; ^2^ Wunambal Gaambera Aboriginal CorporationKalumburuWAAustralia; ^3^ Bush Heritage AustraliaMelbourneVic.Australia

**Keywords:** Australian monsoon tropics, fire, geologic substrates, rainfall gradients, rainforests, topographic fire protection

## Abstract

The small rainforest fragments found in savanna landscapes are powerful, yet often overlooked, model systems to understand the controls of these contrasting ecosystems. We analyzed the relative effect of climatic variables on rainforest density at a subcontinental level, and employed high‐resolution, regional‐level analyses to assess the importance of landscape settings and fire activity in determining rainforest density in a frequently burnt Australian savanna landscape. Estimates of rainforest density (ha/km^2^) across the Northern Territory and Western Australia, derived from preexisting maps, were used to calculate the correlations between rainforest density and climatic variables. A detailed map of the northern Kimberley (Western Australia) rainforests was generated and analyzed to determine the importance of geology and topography in controlling rainforests, and to contrast rainforest density on frequently burnt mainland and nearby islands. In the northwestern Australian, tropics rainforest density was positively correlated with rainfall and moisture index, and negatively correlated with potential evapotranspiration. At a regional scale, rainforests showed preference for complex topographic positions and more fertile geology. Compared with mainland areas, islands had significantly lower fire activity, with no differences between terrain types. They also displayed substantially higher rainforest density, even on level terrain where geomorphological processes do not concentrate nutrients or water. Our multi‐scale approach corroborates previous studies that suggest moist climate, infrequent fires, and geology are important stabilizing factors that allow rainforest fragments to persist in savanna landscapes. These factors need to be incorporated in models to predict the future extent of savannas and rainforests under climate change.

## 1 Introduction

The global extent of closed canopy tropical rainforests and savannas is determined by climate, especially mean annual precipitation (Lehmann et al., 2014; Murphy & Bowman, 2012). However, at around 1,000–2,000 mm/year rainforest and savanna form vegetation mosaics (Hirota, Holmgren, van Nes, & Scheffer, 2011; Staver, Archibald, & Levin, 2011a, 2011b). Tropical savannas are characterized by a low tree cover and a high biomass of C4 grasses, which supports frequent fires in the dry season (Bond, Woodward, & Midgley, 2005; Hoffmann, Jaconis, et al., 2012). By contrast, tropical rainforests have a species‐rich tree flora that form dense canopies, little grass, and infrequent fire activity.

The mechanisms that control patterning of rainforest and savanna mosaics are disputed, with debate polarized between the importance of fire and soils. One view is that edaphic factors like soil nutrients are the main control of rainforest–savanna mosaics, and fire is not a cause but rather a consequence of vegetation patterns (Lloyd et al., 2008; Veenendaal et al., 2015). Although savanna soils may have sufficient nutrient stocks to support rainforest trees (Bond, 2010; Vourlitis et al., 2015), rainforests are generally found on more nutrient‐rich soils compared with savannas (Dantas, Batalha, & Pausas, 2013; Silva et al., 2013). Infertile savanna soils are known to limit expansion of rainforest (Silva et al., 2013), while deeper and more fertile substrates allow rainforest to grow in drier climates (known as “edaphic compensation”; Ash, 1988; Webb, 1968). However, it is not clear whether these patterns result from a direct edaphic effect or from local feedbacks. Soils underneath rainforests are often more rich in nutrients, compared with savannas, regardless of the inherent fertility of soil parent material (Dantas et al., 2013; Silva et al., 2013), because of nutrient acquisition and cycling (Silva et al., 2008). Tree canopy cover and canopy productivity increase soil nutrient content (Paiva, Silva, & Haridasan, 2015), particularly N concentration and availability (Schmidt & Stewart, 2003). Consequently, there are substantial practical difficulties in making ecologically meaningful measurements of soils fertility variation, particularly across rainforest ecotones, where forest boundaries wax and wane (Silva et al., 2013; Warman, Bradford, & Moles, 2013).

The alternative view is that rainforest and savanna are “bi‐stable” in regions with intermediate productivity, and the realization of vegetation depends on landscape fire history (Bond et al., 2005; Dantas, Hirota, Oliveira, & Pausas, 2016; Hoffmann, Geiger, et al., 2012; Murphy & Bowman, 2012; Staver et al., 2011a; Warman & Moles, 2009). This view is based on alternative stable state (ASS) theory whereby stabilizing feedbacks hold rainforest or savanna in specific “basins of attraction” (Hirota et al., 2011). Resolving the role of edaphic factors in controlling rainforest boundaries directly or indirectly via feedbacks is complex and demands multiple lines of evidence, including direct measurements of soils, modeling, and experiments (Bowman, Perry, & Marston, 2015). Analysis of remote sensing estimates of canopy cover at a global scale has been presented as evidence for the bimodal distribution of rainforests and savannas (Staver et al., 2011b). It has been argued that the intensity of the bimodality may be a statistical artifact associated with the use of regression tree (CART) analyses, which impose discontinuities in satellite tree cover estimates (Hanan, Tredennick, Prihodko, Bucini, & Dohn, 2014, 2015; Staver & Hansen, 2015), although global canopy height analyses, based on products derived from LiDAR measurements, confirmed the bimodality detected through satellite data (Xu et al., 2016).

Regional‐level analyses based on remote sensing have been employed in studies investigating the environmental controls of different types of vegetation (Dahlin, Asner, & Field, 2014; Fensham, Fairfax, & Archer, 2005; Murphy et al., 2010). However, there has been surprisingly limited analysis of rainforest–savanna mosaics at a regional level. In an important pioneering study, Ash (1988) synthesized data from topographic maps, aerial photography, and field data to create a model of the environmental controls of rainforests and savanna vegetation in the wet tropics of North Queensland (Australia), to assess the relationship between rainforest location and environmental characteristics. Ash (1988) concluded that the distribution of rainforest boundaries can be empirically predicted based on water availability and topography, and substrate fertility might allow rainforests to expand into otherwise unfavorable environments. This research was supported by Fensham (1995), who employed aerial photography and satellite imagery to investigate the relation between dry rainforest and environmental variables in North Queensland. To the best of our knowledge, there are no other map‐based analyses of rainforest–savanna mosaics at a regional scale anywhere else in the tropics. These rainforest patches are known to be biodiverse and important for a broad cross section of fauna (Price, 2006; Tutin, White, & Mackanga‐Missandzou, 1997), yet they have been poorly researched compared with the more extensive wet rainforests (Sánchez‐Azofeifa et al., 2005).

Northwestern Australia is an attractive model system because it spans a wide rainfall gradient at the driest extreme of the Australian tropical rainforest estate (Bowman, 2000). The global analysis of Staver et al. (2011b) suggests the region is deterministically savanna; yet, tiny patches of rainforest exist, embedded in the savanna matrix. These environments rainforests are more exposed to fire due to their higher boundary/core ratio; nonetheless, in some locations rainforest expansion has occurred (Banfai & Bowman, 2006; Bowman, Walsh, & Milne, 2001; Clayton‐Greene & Beard, 1985). Studies from northern Australia and elsewhere in the tropics have identified the importance of landscape setting in determining rainforest distribution in areas subject to high fire activity. For example, rainforests can be more abundant on islands that have lower fire activity than adjacent mainland savannas (Clayton‐Greene & Beard, 1985). Rainforests can also be confined to steep gullies or valleys (Bowman, 2000; Ibanez et al., 2013; Warman & Moles, 2009) because of the fire protection they provide (Murphy & Bowman, 2012), although additional effects of higher nutrient and water availability could also be important (Ash, 1988).

We employed a macroecological approach to determine the effect of climatic and geomorphological factors (topography and geology) on rainforest abundance at a large spatial scale. Geology was used as a proxy for the nutrient stock provided by the parent material, to exclude the effect of vegetation on soil fertility. To assess the correlations between climate and rainforest distribution in the entire northwestern Australian monsoon tropics, we analyzed existing subcontinental‐scale vegetation maps. We then assessed the importance of topography and geology at a regional scale, as the effects of these factors on rainforest distribution are detectable at this scale, compared with climate (Murphy & Bowman, 2012). To do so, we generated a detailed map of rainforests in the northern Kimberley (Western Australia), which is characterized by a limited rainfall range (200 mm/year), and a variety of geologies and topographic settings. Within this region, we undertook a local‐scale “natural experiment” comparing the influence of topography and fire activity on rainforest density on mainland and adjacent islands with similar rainfall, geology, and distance from the coastline. We addressed the following hypotheses:

- At a subcontinental scale, factors associated with water availability are the main climatic drivers determining rainforest density;
- At a regional scale, topography and geology affect rainforest distribution;
- At a local scale, the importance of insularity and topography is directly related to fire activity. In locations with high fire activity (mainland), rainforests are predominantly confined to fire‐sheltered settings, whereas in areas with lower fire activity (islands), rainforests will also be able to grow in more exposed settings.

Collectively this study investigates the drivers of rainforest distribution across multiple spatial scales in northern Australia, thereby illuminating the capacity for climate change and fire management to affect rainforest coverage and providing insights for both theoretical ecology and applied land management.

## 2 Methods

### 2.1 Geographic context

**Figure 1:**
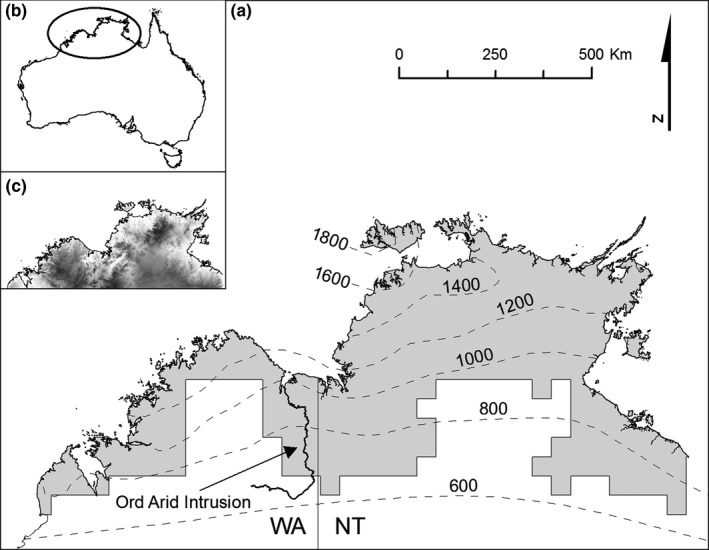
The monsoon rainforest domain in northwestern Australia. (a) The gray area represents the monsoon rainforest domain in the north of Western Australia (WA; Kimber et al., 1991) and the Northern Territory (NT). The Ord Arid Intrusion, the main biogeographic barrier between the two states, is indicated. Dashed lines indicate rainfall isohyets (mm). The insets show (b) the study area within Australia and (c) elevation (minimum, 0 m, white; maximum, 960 m, black)

**Figure 2:**
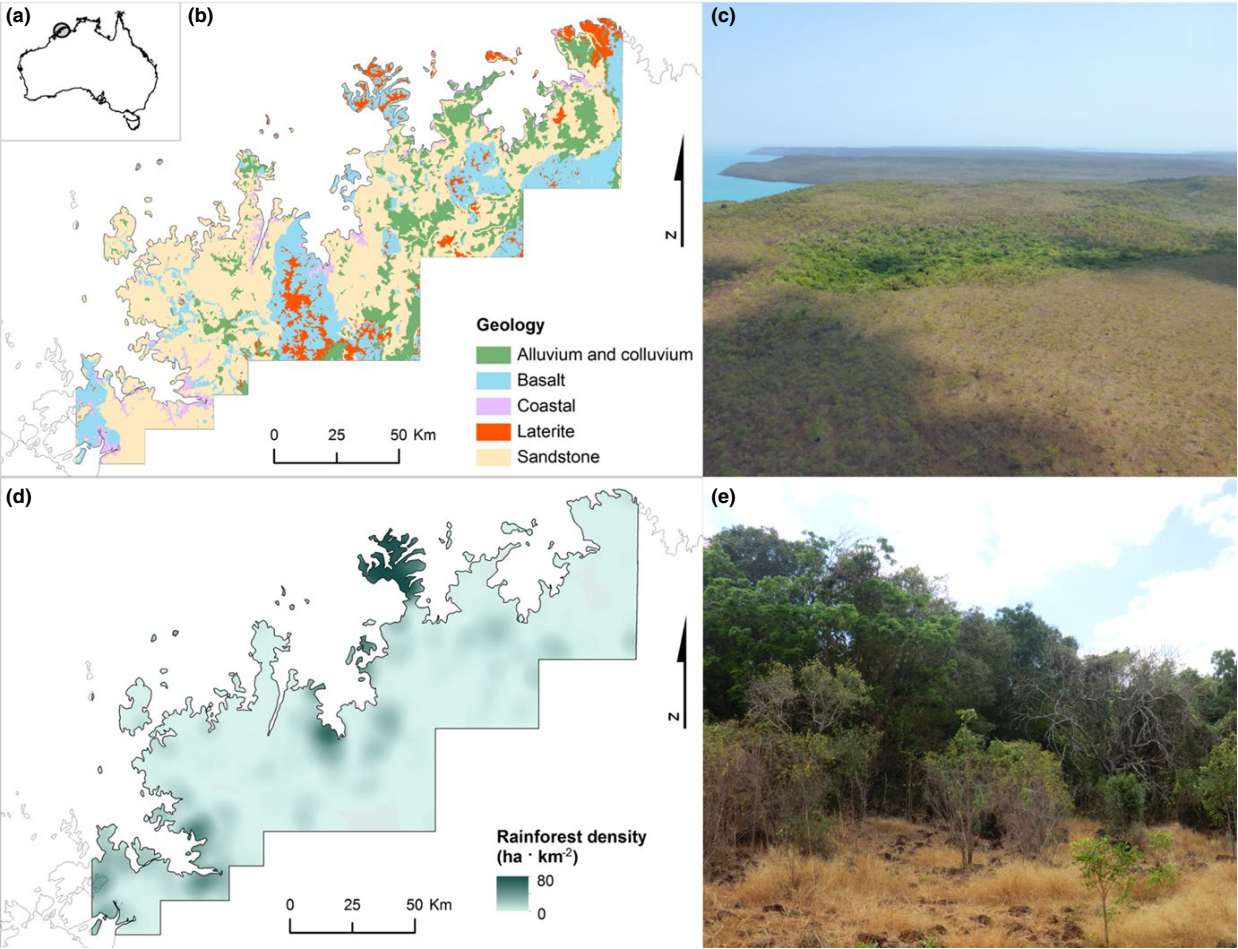
(a) Extent of the mapped area in the northern Kimberley; (b) the main geology types in the area, and (d) rainforest density, expressed as ha per km^2^ of land. In this area, rainforests typically occur as (c) small patches (green) surrounded by savanna (brown), with (e) sharp boundaries between the two vegetation types

The Australian monsoon tropics are characterized by a pronounced wet and dry seasons associated with the Australian summer monsoon (Bowman et al., 2010). This region includes the whole of northern Australia except the Australian wet tropics in North Queensland (Bowman, 2000; Figure 1a‐c). In contrast to the wet tropics, where tropical rainforests dominate, the monsoon tropics support vast eucalypt savannas (Bowman, 2000; Figure 2a,c). Embedded in these savannas are very small patches of monsoon tropical rainforest, ranging from a few trees to 100 ha in area (McKenzie, Belbin, Keighery, & Kenneally, 1991). These rainforests have floristic and biogeographic affinities with wet tropical rainforests in both Asia and Australia. They have been intensively studied given their unusual biogeography and ecology, particularly their ability to persist in a highly flammable tropical savanna environment (Bowman, 2000). Some rainforests are known to grow on aquifers (Kenneally, Keighery, & Hyland, 1991; Russell‐Smith, 1991), which insulate the patches from regional climate, but our mapping could not differentiate these types from the more widespread and drought‐adapted rainforests (Bowman, Wilson, & McDonough, 1991; Russell‐Smith, 1991). The locus of the subcontinental study was the Australian monsoon tropics west of the Carpentarian Gap biogeographic divide, which separates the biota of the Northern Territory and Western Australia from Cape York Peninsula (Bowman et al., 2010). Annual rainfall in this area varies from approximately 1,900 mm in the northeast to 700 mm in the southwest (Figure 1c), that would be expected to exert a strong influence on the abundance of rainforest. This analysis was made possible by combining vegetation maps produced by the Northern Territory and Western Australian government land management agencies, noting that the border between the two states broadly align with the Ord Arid Intrusion, a major biogeographic boundary that separates the biota of the Kimberley region of Western Australia from that of the “Top End” of the Northern Territory (Figure 1; Bowman et al., 2010; Eldridge, Potter, & Cooper, 2011), and that likely affects rainforest species diversity. In addition to this coarse‐scale subcontinental study, we undertook a more detailed analysis of the rainforests to the west of the Ord Arid Intrusion. This region, located at the extreme end of the precipitation gradient where rainforest occurs in northern Australia, has limited spatial variability in rainfall (1,200–1,400 mm), which allowed us to identify ecological factors, other than precipitation, that shape rainforest distribution. This was based on fine‐scale mapping of the traditional lands of the Wunambal Gaambera people, henceforth called the Wunambal Gaambera Country.

The Wunambal Gaambera Country occupies an area of 9,144 km^2^, dominated by biodiverse tropical savannas occurring on deeply weathered sandstones and basaltic base rocks of Precambrian age, often capped by Cainozoic laterites. In this region, average annual rainfall occurs almost entirely during the summer wet season (November–April), while the rest of the year is almost rain‐free (Beard, 1976). The landscapes are shaped by geology; the dominant substrates are infertile sandstone, where the Holocene sea‐level rise has created rugged coastlines, and the moderately fertile basalt country, characterized by gentle slopes and hills (Beard, 1979; Speck et al., 2010; Figure 2b). The vegetation is predominantly eucalypt savanna. *Eucalyptus tetrodonta*–*Eucalyptus miniata* savannas are found on the laterite mesas and hills, while *Eucalyptus tectifica*–*Eucalyptus grandifolia* savannas are common on deeper, clay soils on plains. Small patches of semi‐deciduous rainforests are interspersed in the savanna (Figure 2c,e), typically located in fire‐protected locations (Vigilante, Bowman, Fisher, Russel‐Smith, & Yates, 2004).

Fire regimes in the northern Kimberley are strongly shaped by anthropogenic ignitions and have been for over 40,000 years (O'Connor, 1995). This ancient tradition of Aboriginal fire management is likely to have maintained biodiverse open savanna habitats and protected small isolated rainforest fragments (Mangglamarra, Burbidge, & Fuller, 1991; Trauernicht, Brook, Murphy, Williamson, & Bowman, 2015; Vigilante, Murphy, & Bowman, 2009). The cessation of Aboriginal fire management in many northern Australian environments has been associated with degradation of some rainforests and other fire‐sensitive plant communities (Russell‐Smith & Bowman, 1992; Trauernicht, Murphy, Portner, & Bowman, 2012), although in rarely burnt areas there can be expansion of rainforest (Bowman & Fensham, 1991; Clayton‐Greene & Beard, 1985).

### 2.2 Rainforest mapping and analyses

#### 2.2.1 Subcontinental scale—climatic drivers of rainforest density

The distribution and areal extent of the rainforests in the northwestern Australian monsoon tropics was determined by blending existing vegetation maps. Total coverage of rainforest in Western Australia and Northern Territory was calculated for a lattice of grid cells 50 × 50 km in area. The Western Australia map (1:200,000) was derived from Kimber, Forster, and Behn (1991), who used semi‐automated classification of Landsat imagery taken in 1986 and did not differentiate floristics or structure variation among rainforests. The Northern Territory vegetation data (1:80,000), based on interpretation of aerial photography classified according to Russell‐Smith (1991), were supplied by the Department of Land Resource Management, © Northern Territory of Australia. In calculating rainforest coverage in the Northern Territory lattice cells, we selected both dry and wet rainforest types because they are structurally and floristically similar to the Western Australian rainforests (Bowman, 1992; Kenneally et al., 1991). We combined the Western Australia and Northern Territory data to create a map of the northwestern rainforest domain, extending from 11.00°S to 18.00°S in latitude and from 122.14°E to 138.00°E in longitude (Figure 1). This resulted in 192 and 63 grid cells in the Northern Territory and Western Australia, respectively. For each grid cell, mean annual rainfall, precipitation seasonality (coefficient of variation of monthly rainfall expressed as a percentage), potential evapotranspiration, moisture index (mean annual precipitation over potential evapotranspiration; Thornthwaite, 1948), and annual mean temperature were calculated for the center point of each cell. Rainfall, precipitation seasonality, and temperature data were obtained from WorldClim Global Climate Data (Hijmans, Cameron, Parra, Jones, & Jarvis, 2005), and moisture index and potential evapotranspiration were downloaded from the Global Aridity and PET database (Zomer, Trabucco, Bossio, & Verchot, 2008). Minimum, maximum, and median values of the climatic variables were calculated separately for the Western Australia and Northern Territory grid cells.

#### 2.2.2 Regional scale—regional drivers of rainforest density

We generated a map of the rainforests in the northern Kimberley, covering the entire Wunambal Gaambera Country and expanding the analysis to the adjacent coastal areas (total surface 12,572 km^2^), as follows. Orthophotos (scale 1:8,000) taken during the dry season (May–August) of the years 2005–2007 were used create a map of the rainforest patches located in the study area. A lattice of 30 × 30 m cells was overlaid on the orthophotos, and every cell was manually classified as “rainforest,” “savanna,” or “other.” The vegetation type of each cell was considered to be the one occupying the highest proportion of the cell. A map of the rainforest patches was produced by merging the contiguous cells classified as “rainforest” (Figure 2d). A helicopter survey was conducted to validate the map. The flight path, designed to include locations with both high and low rainforest cover, included coastal and inland areas as well as islands. It covered the main geologic substrates, in particular basalt, sandstone, and laterite. We flew along the selected path at an average height of 300 m above the ground for a total length of 550 km on 22 September 2013. Waypoints, collected every 10 s, were visually identified as “rainforest” or “savanna.” The points were then buffered 30 m and intersected with the rainforest map. A confusion matrix was generated to calculate map accuracy, omission and commission errors, and kappa coefficient of agreement (Congalton, 1991).

Patch size, distance from the coastline, and distance from the nearest drainage line were calculated for every rainforest polygon on the regional scale map. Rainforest density was calculated as ha of rainforest per km^2^ of land, based on a grid of 1 × 1 km size cells for computational reasons. For each cell, we also calculated: (1) the geology category, based on the predominant geology type in each 1 × 1 km cell; and (2) the topographic category, based on the predominant topographic position index (TPI; Jenness, 2006) in the cell. The TPI was calculated for every pixel in the mapped area from a 30‐m resolution digital elevation model (DEM), based on the difference in elevation between each pixel and the average elevation of the eight neighboring pixels; values lower than −1 were classified as “valley,” and values higher than +1 as “ridge.” Intermediate values were classified as “flat” or “slope” depending on the slope of the pixel (≤4° for flat areas, >4° for slopes), obtained from the 30‐m DEM. (3) Each grid cell was further classified as having “complex” or “level” terrain, noting that complex terrain is often associated with rockiness. Cells in which the categories “valley” + “ridge” + “slope” occupied more than 50% of the cell were classified as “complex,” the others as “level,” The average rainforest density in the northern Kimberley was then calculated for each geologic substrate and TPI based on the rainforest density grid.

#### 2.2.3 Local‐scale natural experiment–Mainland versus Islands

We expected there would be differences in fire activity and rainforest distribution between islands and mainland, because islands have been subject to fewer human ignitions due to infrequent visitation in recent times (Vigilante et al., 2013) and the sea provides a natural fire break from surrounding landscape fires. To test this, we compared rainforest density grid cells on islands and the mainland. We selected areas that were geographically, floristically, and ecologically similar by extracting from the “regional scale dataset” only grid cells with the following attributes: mean annual rainfall between 1,250 and 1,382 mm/year, distance from the coastline <5 km (equivalent to the radius of the biggest island hence the maximum distance from the coastline on islands), and geology developed on basalt, laterite, or coastal sediments. Islands and coastal areas of the northern Kimberley are floristically similar, with only a very small group of taxa recoded only from islands (Lyons, Keighery, Gibson, & Handasyde, 2014). Grid cells located on the Bougainville Peninsula were included in the category “islands,” due its narrow neck which makes it functionally equivalent to an island in terms of isolation from the mainland.

Fire activity was calculated from a 15‐year fire history map (2000–2014), created at a pixel resolution of 250 m based on MODIS satellite imagery, accessed via North Australian Fire Information website (http://www.firenorth.org.au/nafi3/). Due to the coarse resolution of the fire history map, it was impossible to accurately locate every fire scar, so the data were used to provide coarse‐scale information about differences in fire activity between the mainland and islands. For every cell of the rainforest density grid, the area‐weighted proportion of years burnt was calculated by dividing the average number of years in which the cell was burnt by 15, the total number of years investigated. The average fire activity per year and rainforest density were calculated for cells classified as “island” or “mainland” and, within each category, “complex” or “level” terrain.

### 2.3 Statistical analyses

At a subcontinental scale, we employed the Pearson product moment correlation coefficient to examine correlations among rainforest density and the climatic variables, and presented the results in a constellation diagram. For presentation (but not the analysis), we aggregated the grid cells into 200‐mm mean annual rainfall bins and calculated the average rainforest density for each bin.

At a regional scale, we first tested for spatial autocorrelation in rainforest density and assessed minimum sampling distance, estimated by plotting the semi‐variance as a function of distance, using the software R (R Core Team, 2013) and the R package geoR (Ribeiro & Diggle, 2001; Appendix S1). We then tested whether the factors terrain and geology are related to rainforest density. We also checked whether rainforest density was associated with geology within level and complex terrain types. To do this, we used generalized linear models (GLMs) and complete subset regression and model selection based on Akaike's information criterion (AIC; Burnham & Anderson, 2002), calculated using the R package “MuMIn” (Bartoń, 2009). We used the compound Poisson‐gamma distribution, included in the tweedie family of distributions, which allows regression modeling of zero‐inflated positive continuous data (R packages “tweedie” [Dunn, 2014] and “statmod” [Smyth, Hu, Dunn, Phipson, & Chen, 2015]). To assess the importance of each variable, we calculated Akaike weights (*w*_i_), which represent the probability that a given model is the best in the candidate set (Burnham & Anderson, 2004). We then calculated variable importance (*w*+) as the summed *w*_i_ of the models in which the variable occurs. *w*+ values higher than 0.73 were considered to indicate that the variable is a statistically important predictor (Murphy et al., 2010). Model summaries are provided in Appendix S2.

When comparing mainland versus islands, we examined differences in rainforest density and fire activity between locations, testing for the factors insularity (island or mainland) and terrain (complex or level). To do so, we employed GLMs, using the compound Poisson‐gamma distribution for both rainforest density and fire activity, complete subset regression, and model selection based on AIC as described above. Variable importance was assessed by calculating *w*+, as described above. Model summaries are provided in Appendix S2.

## 3 Results

### 3.1 Subcontinental scale

**Figure 3:**
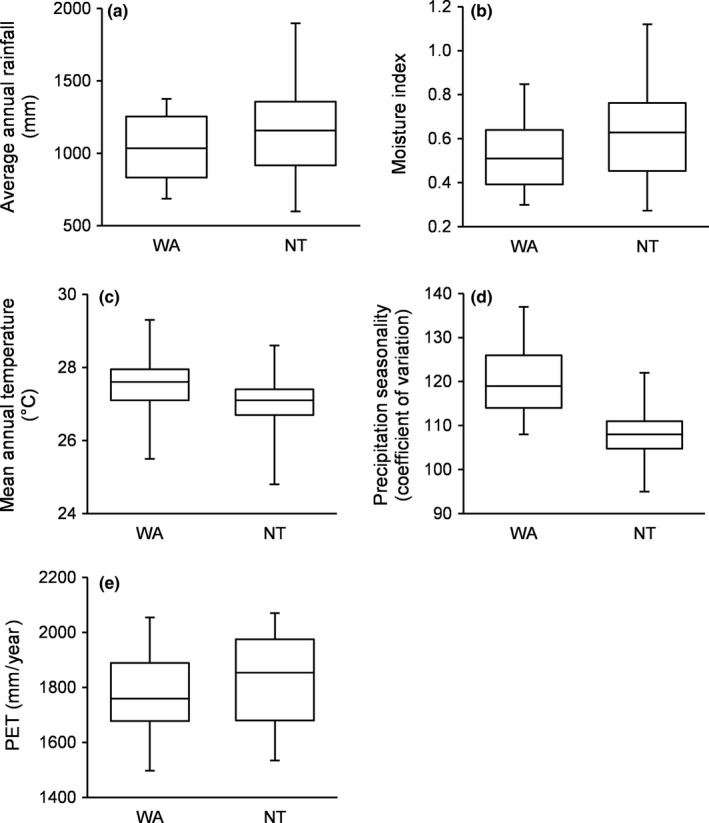
Comparison of the rainforest domain in Western Australia (WA) and Northern Territory (NT), showing (a) average annual rainfall, (b) moisture index, (c) mean annual temperature, (d) precipitation seasonality, and (e) potential evapotranspiration (PET). Boxes indicate median values and upper and lower quartiles, bars the 10th and 90th percentiles

The northwestern Australian rainforest domain covered an area of 640,000 km^2^, ranging from the coastline to a maximum of 350 km inland. Rainforest density was lower west of the Ord Arid Intrusion: in Western Australia rainforest density ranged from 0 to 8.7 ha of rainforest per km^2^ of land (average 1.1 ± 0.2 ha/km^2^), while in the Northern Territory the range was 0–19.0 ha/km^2^ of land (average 1.4 ± 0.2 ha/km^2^). The Northern Territory showed higher median values and a broader range of both mean annual rainfall and moisture index (Figure 3a,b). Mean annual temperature and precipitation seasonality showed higher median and maximum values in Western Australia and minimum in the Northern Territory (Figure 3c,d), while annual potential evapotranspiration had a similar range in the two states but higher median values in the Northern Territory (Figure 3e).

**Figure 4:**
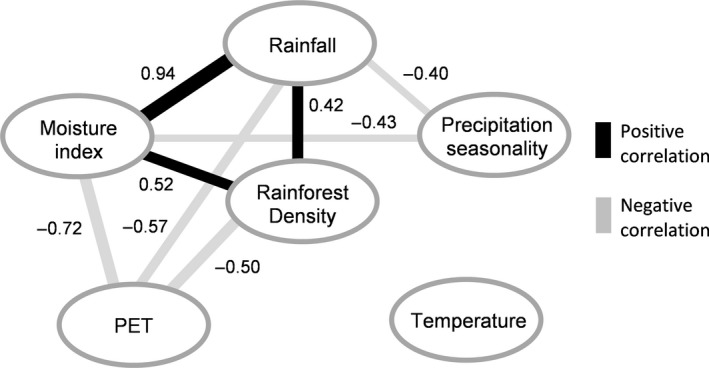
Constellation diagram showing the strength and direction of correlations among rainforest density and the climatic variables average annual rainfall, moisture index, potential evapotranspiration (PET), precipitation seasonality, and mean annual temperature in the monsoon rainforest domain in northwestern Australia. Positive correlations are represented by black lines, negative correlations by gray lines. Correlations stronger than .4 or −.4 are indicated; wider lines indicate stronger correlations, narrower lines weaker correlations

**Figure 5:**
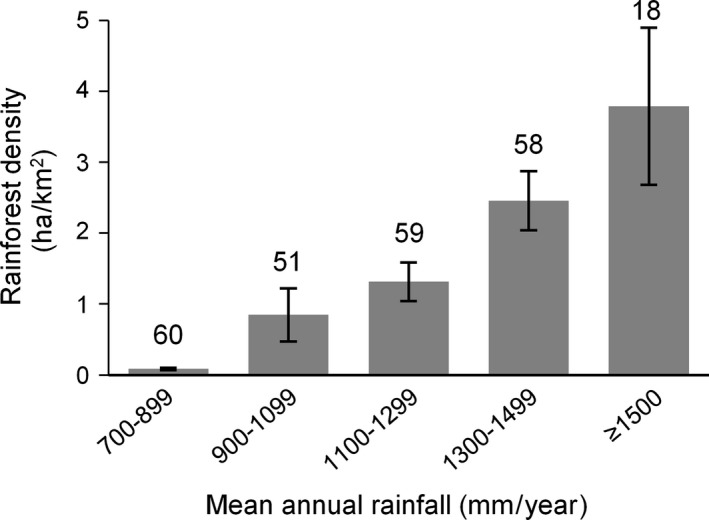
Average rainforest density by average annual rainfall, calculated within the rainforest domain in the Australian monsoon tropics. The numbers above each column represent the number of grid cell included in that rainfall interval. Error bars represent standard error

There was a positive correlation between rainforest density and both mean annual rainfall and moisture index (Figures 4 and 5), which were also positively correlated. Potential evapotranspiration was negatively correlated with rainforest density, moisture index, and rainfall, while precipitation seasonality was negatively correlated with rainfall and moisture index. Mean annual temperature was not correlated with any of the climatic variables investigated nor with rainforest density.

### 3.2 Regional scale

In total, 2,902 points were assessed during the aerial survey. There was a strong concordance between the rainforest map and the aerial assessment, with a resulting overall map accuracy of 93% (Kappa coefficient .78; Appendix S3). A high accuracy was obtained for savanna points (95% for both producer's and user's accuracy), meaning few savanna points were mistaken for rainforest. We attribute the lower producer's and user's accuracy for rainforests (83% and 82%, respectively) to the floristic composition of the monsoon vine thickets, where semi‐deciduous species can dominate (Beard, 1979), making portions of the forest patches undetectable from orthophotos taken during the dry season.

**Figure 6:**
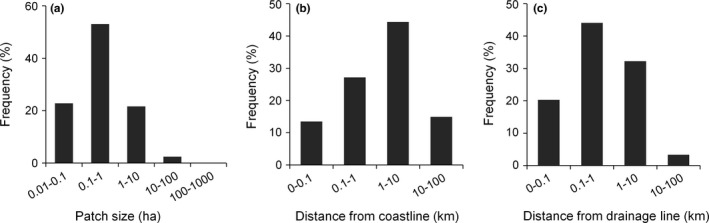
Frequency distribution of rainforest patches in the northern Kimberley region according to (a) size, (b) distance from the coastline, and (c) distance from the nearest drainage line. Note the logarithmic scale for the *x* axes

Savanna was by far the most common vegetation, covering 98.9% of the area. We detected a total of 6,460 rainforest patches covering 10,300 ha, equivalent to 0.82% of the mapped land. Patch size ranged from 0.1 to 220 ha and averaged 1.6 ha ± 0.1 (*SE*). Seventy‐five percent of patches were smaller than 1 ha, and only 2.5% were larger than 10 ha (Figure 6a). More than 40% of the mapped rainforest patches were located within 1 km of the coastline (Figure 6b), but patches were detected up to 47 km inland (average 4.7 km ± 0.1 [*SE*]). A similar pattern was identified for distance from drainage lines, with 64% of the patches located within 1 km of the nearest drainage line (Figure 6c), but some up to 32 km distant (average 1.7 km ± 0.0 [*SE*]).

**Figure 7:**
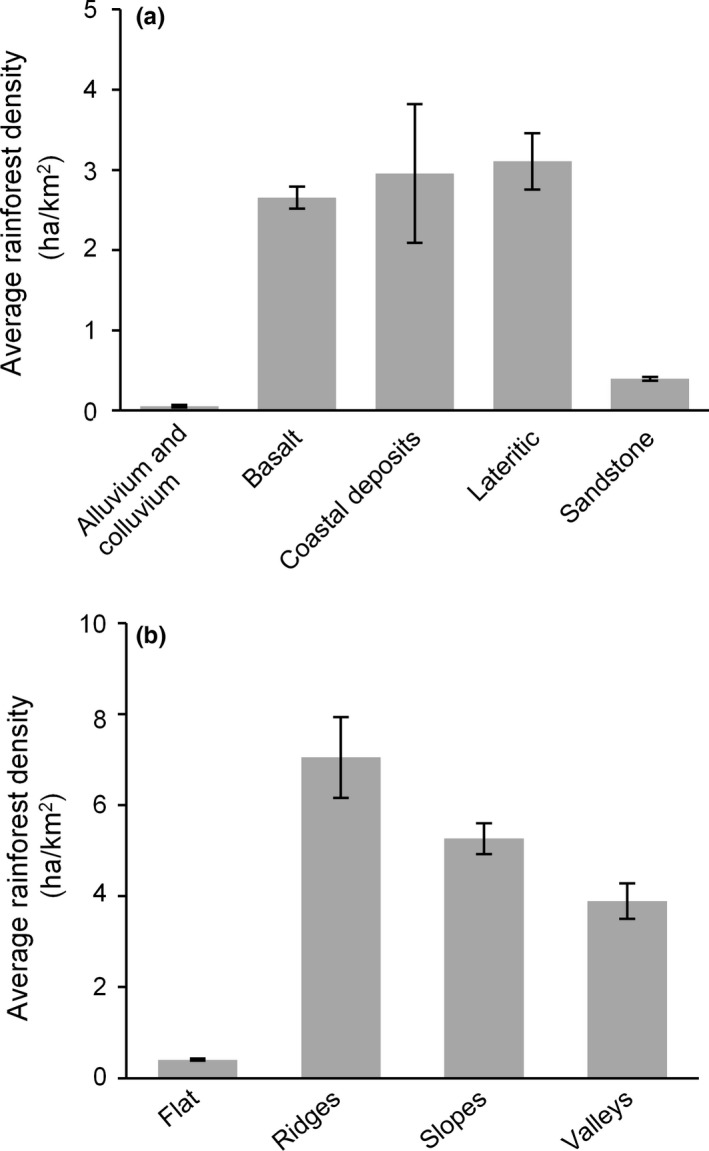
Rainforest density in relation to (a) geologic substrate, and (b) topographic position in the northern Kimberley (regional analysis). Rainforest density was highest on coastal sediments, basalt, and lateritic substrates. It was also higher on ridges, slopes, and valleys, and almost absent in flat areas. Error bars represent standard errors

Rainforest density was strongly dependent on both terrain (*w*+ = 1.00) and geology (*w*+ = 1.00); average rainforest density was higher on relatively fertile substrates (laterite, coastal sediments, and basalt), and lower on alluvium and colluvium and infertile sandstone (Figure 7a). Average rainforest density was also higher in complex terrain such as ridges, slopes, and valleys and lower on level areas (Figure 7b). The model including geology and terrain explained 32.1% of the deviance. The preference for relatively nutrient‐rich geology was independent on terrain, as on both level and complex terrains rainforest density was strongly associated with geology (*w*+ = 1.00 in both cases; Appendix S4). Geology explained 13% of deviance on complex terrain and 10% of deviance on level terrain.

### 3.3 Mainland versus Islands

The grid cells on basalt, laterite, and coastal substrates and within 5 km of the coast covered an area of 332.4 km^2^ on islands and 693.1 km^2^ on the mainland. The total area covered by rainforests was 47.0 km^2^ on the islands, compared with 13.6 km^2^ on the mainland, so that rainforest density was seven times higher on the islands (Table 1). Islands were more topographically complex than the mainland (Table 1). There was statistical support for an influence of both insularity (*w*+ = 1.00) and terrain (*w*+ = 1.00) on rainforest density (Table 2), and the model including both explained 35% of deviance. There was less fire activity on islands (average 0.061 ± 0.003 times burnt per year) than on the mainland (average 0.266 ± 0.004 times burnt per year; *w*+ = 1.00), and insularity alone accounted for 39.1% of deviance. Contrary to expectations, there was no statistical support for an effect of terrain on fire activity (*w*+ = 0.48; Table 2).

**Table 1:** Extent, rainforest cover, average rainfall, and extent of the geologic substrates and terrain type on the selected grid cells used to compare rainforest density on islands and the mainland in the northern Kimberley

	Islands	Mainland
Total land (km^2^)	332.4	693.1
Rainforest area (km^2^)	47.0	13.6
Rainforest density (ha/km^2^)	14.1	2.0
Average rainfall (mm/year)	1348 ± 1	1307 ± 1
Geology type (%)		
Basalt	64.4	81.2
Coastal sediments	0.5	2.7
Laterite	35.1	16.1
Terrain type (%)		
Complex	62.8	55.6
Level	37.2	44.4

**Table 2:** Average fire activity, measured as times burnt per year, and average rainforest density, measured as ha/km^2^, for complex and level terrains located on the selected grid cells on islands and mainland in the northern Kimberley

	Level terrain		Complex terrain		*w+*	
	Mainland	Islands	Mainland	Islands	Terrain	Insularity
Fire activity (average times burnt per year ± *SE*)	0.26 ± 0.01	0.08 ± 0.01	0.27 ± 0.01	0.05 ± 0.00	0.48	1.00
Rainforest density (ha/km^2^ ± *SE*)	0.30 ± 0.06	6.81 ± 0.73	4.29 ± 0.42	19.69 ± 0.88	1.00	1.00

The *w+* indicates the statistical support for the terms terrain and insularity when comparing mainland and islands (full results are presented in Appendix S2).

## 4 Discussion

We found that in northwestern Australian monsoon tropics rainforest patches are tiny and scattered across a vast savanna matrix. Due to their small size, these rainforest fragments are essentially undetectable at the resolution employed by global‐level assessments (Murphy & Bowman, 2012; Staver et al., 2011b). At a subcontinental scale, the strong correlation between rainforest density and annual rainfall, potential evapotranspiration, and moisture index highlighted the primacy of water supply compared with mean annual temperature and precipitation seasonality, and supported our first hypothesis. This correlation is congruous with the observation that a trend of increasing rainfall in northern Australia since the 1940s is the key driver of rainforest patch expansion (Banfai & Bowman, 2007; Bowman et al., 2001). These findings are also consistent with the global trend of increasing proportion of rainforest (and decreasing savanna) as mean annual precipitation increases (Hirota et al., 2011; Murphy & Bowman, 2012). In the drier landscapes west of the Ord Arid Intrusion, rainforest species diversity is also lower than to the wetter east and most of the species in Western Australia are as subset of those in the Northern Territory (Kenneally et al., 1991). However, the presence of rainforests in the northwestern Australian monsoon tropics showed that the region cannot be defined as deterministically savanna based solely on climate (Murphy & Bowman, 2012). Similarly, rainforest patches exist throughout much of the tropics globally, which suggests that in all but arid tropical regions, climate alone is not the only factor controlling rainforest distribution (Staver et al., 2011b). In Brazil, for example, small patches of deciduous and semi‐deciduous rainforests are interspersed in a matrix of savanna plants (Cerrado) or thorn scrubs (Caatinga) and are restricted to slopes and moist, nutrient‐rich sites (Leal, da Silva, Cardoso, Tabarelli, & Lacher, 2005; Oliveira‐Filho & Ratter, 2002). Likewise, in Ivory Coast the dominant savanna vegetation is scattered with small patches of dry rainforest (Goetze, Hörsch, & Porembski, 2006).

We also found support for our second hypothesis that topography and geology affect rainforest distribution. The influence of topography on rainforest density was manifest in the higher rainforest density on complex compared with level terrain. Rainforest density was also higher on nutrient‐rich basalt compared with the nutrient‐poor sandstone, despite the higher fire frequency and intensity recorded on basalt (Vigilante et al., 2004). This pattern is consistent with the edaphic compensation hypothesis (Ash, 1988; Webb & Tracey, 1981), whose underlying mechanism is probably the effect of increased fertility in enhancing plant growth, allowing trees to reach the threshold size that triggers the switch from savanna to rainforest through grass shading (Hoffmann, Geiger, et al., 2012; Murphy & Bowman, 2012). It is important to note that geology and terrain are typically not independent. For example, in the northern Kimberley rounded hills are more common on basalt, while steep gorges are frequently found on sandstone (Vigilante et al., 2004). However, in our analysis we were able to demonstrate an effect of geology alone by comparing areas on the same terrain, showing that there are more rainforests on basalt than on less fertile geologies.

There was only partial support for our third hypothesis that insularity and topographic effects are directly related to fire activity. Clearly, there were more rainforests on islands, where there was also less fire activity compared with the mainland. The importance of fire in restricting rainforests has been demonstrated by rainforest expansion in other savanna landscapes where fire has been excluded, in northern Australia (Fensham & Butler, 2004; Scott et al., 2012) and elsewhere (Bond, Midgley, Woodward, Hoffman, & Cowling, 2003). Rainforest species are typically less fire tolerant than savanna species due to thinner bark and less developed post‐fire recovery mechanisms (Lawes, Midgley, & Clarke, 2013; Ondei, Prior, Vigilante, & Bowman, 2015; Pausas, 2015). However, we failed to detect a corresponding difference in fire activity between terrain types on the mainland and found only a minor difference on islands. There are two possible reasons for this lack of correspondence, which are not mutually exclusive. One is that terrain, or associated rockiness, did exert an influence on fire activity, but this was obscured by the coarse scale of the grid cells in our analysis (1 × 1 km). Another possible reason is that the higher rainforest density on complex terrain is the result of water and nutrient accumulation (Ash, 1988; Daws, Mullins, Burslem, Paton, & Dalling, 2002), rather than topographic fire protection. Nonetheless, the presence of rainforest on level terrain on islands, but not on the mainland, suggests that fire is an important controller of rainforest distribution in the region.

**Figure 8:**
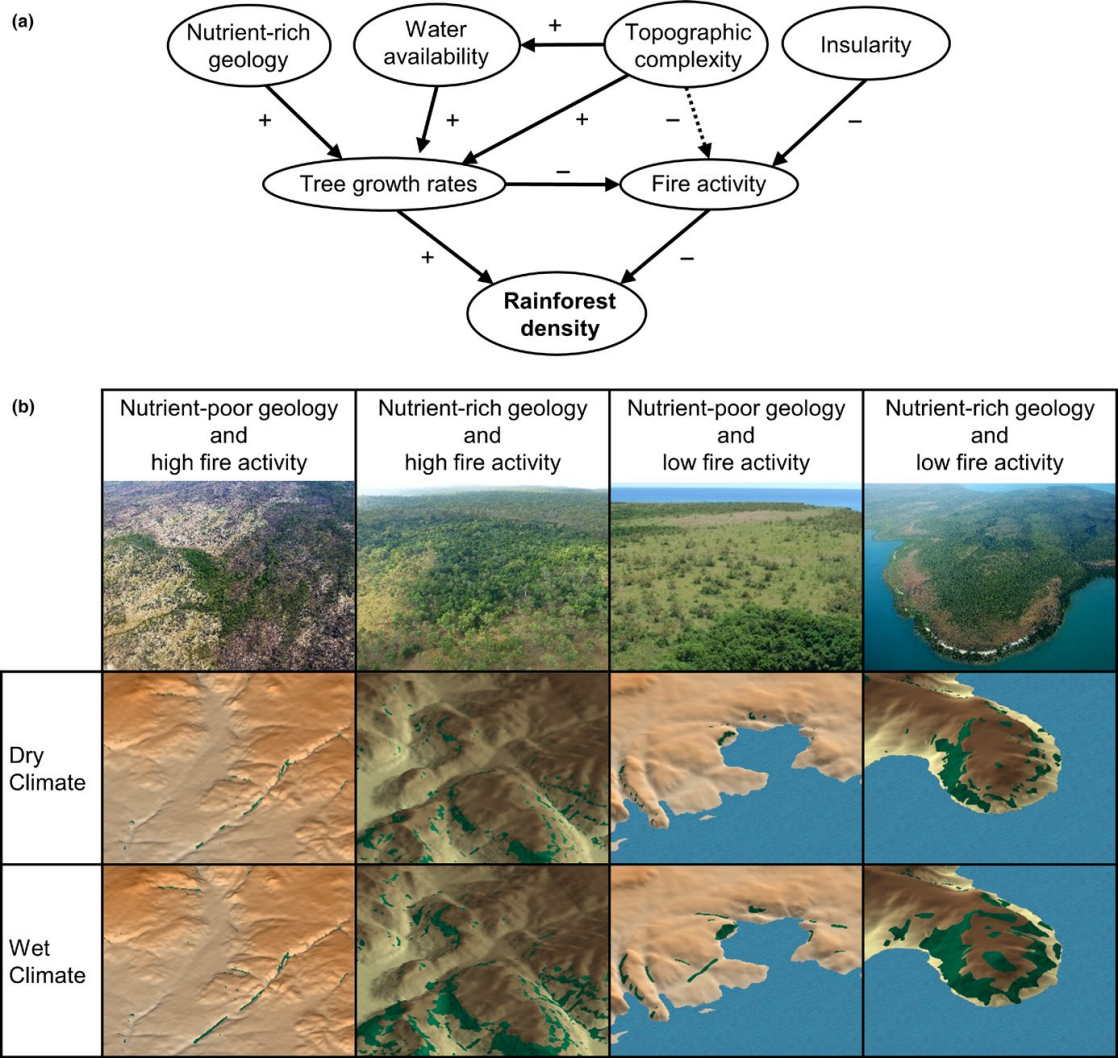
Synthesis of the environmental determinants of rainforest fragments in the northern Kimberley. (a) Diagram showing the positive (+) and negative (−) effects of environmental factors on rainforest density. Dotted lines represent probable effects. (b) Top row: oblique aerial photographs showing examples of the density of rainforest fragments on sites with contrasting geology (sandstone vs. basalt) and fire activity in the northern Kimberley. Second row: 3D renderings of rainforest distribution (dark green) on sandstone (nutrient‐poor) and basalt (nutrient‐rich) landscape under current dry climate. Bottom row: 3D renderings of plausible rainforest density under a climate as wet as coastal regions of the Northern Territory. In this study, we demonstrated that insular sites have substantially lower fire activity than environmentally comparable mainland savanna areas. Under the current climate, rainforest density is highest on fertile infrequently burnt areas, and in frequently burnt landscapes is confined to topographically fire‐protected settings, particularly on nutrient‐poor geology. Under a wetter climate, we expect the rainforest patches to expand and new patches to establish in suitable landscape niches, with the greatest expansion on basalt landscapes. The exact amount of expansion is unpredictable because of the influence of fire activity and fire management

We suggest that rainforest density is determined by the interplay of fire activity and plant growth rates (Figure 8a). Fire activity is shaped by insularity and possibly terrain complexity, while plant growth rates are known to be controlled by water availability and the nutrient stock provided by the geological substrate control (Murphy & Bowman, 2012), with an effect of terrain in enhancing water and soil accumulation (Ash, 1988). Growth rates affect the capacity of rainforest trees to grow rapidly and escape the “fire trap,” thereby developing a closed canopy which shades out grass biomass, reducing fire frequency which in turn reinforces rainforest expansion (Dantas et al., 2013; Hoffmann, Geiger, et al., 2012; Murphy & Bowman, 2012). Our findings are summarized in Figure 8b, which shows characteristic patterns of rainforest fragments in the landscape and how these fragments are likely to expand in response to a wetting climate under contrasting fertility and fire regimes. Rainforest expansion should be proportionally greater in lower‐rainfall areas that currently have low rainforest density, like the northern Kimberley, because there are more landscape niches available for occupancy, such as nutrient‐rich and fire‐protected sites. A prediction of our work is that, under the current wetting trend, there will be continuing rainforest expansion in the Kimberley, as has been observed elsewhere in the Australian tropics (Russell‐Smith, Stanton, Edwards, & Whitehead, 2004).

## 5 Conclusion

We have shown that the density of monsoon rainforest in the northwestern Australian savanna is affected by moisture availability, substrate, and fire. The effect of these drivers appears to involve complicated feedbacks and interaction, such as the combined effects of potential fire protection and increased productivity in topographically complex terrains. We acknowledge that our correlative analysis cannot separate cause and effect, or test the fire‐driven ASS model in explaining the distribution of rainforests. To do this demands analysis of vegetation boundary dynamics coupled with contrasts of substrate type and fire history. This can be achieved through carefully designed regional‐scale analysis of rainforest boundaries trends, such as field surveys and historical sequences of aerial photography (Banfai & Bowman, 2006; Butler et al., 2014; MacDermott, Fensham, Hua, & Bowman, 2016) and is the subject of a subsequent paper. Despite its limitations, our approach is an important step in understanding the effect of climate change and anthropogenic disturbances on naturally fragmented rainforests elsewhere in the tropical savanna biome.

## Conflict of Interest

None declared.
