# Supplementary material for: Water, land, fire, and forest: Multi‐scale determinants of rainforests in the Australian monsoon tropics
Source: Ecol Evol. 2017 Feb 9;7(5):1592–604. doi: 10.1002/ece3.2734 (PMC5330914; doi:10.1002/ece3.2734)
Supplement: Supplementary file 1 [file ECE3-7-1592-s001.docx]

# Appendix

**Water, land, fire and forest: multi-scale determinants of rainforests in the Australian monsoon tropics**

Stefania Ondei, Lynda D. Prior, Grant J. Williamson, Tom Vigilante and David M.J.S. Bowman

**Appendix 1.** Semi-variogram describing the spatial pattern of rainforest density observations, obtained from the north Kimberley rainforest map. To measure the degree of spatial dependence between samples we plotted the semi-variance as a function of distance between pairs of observations. The graph shows that the semi-variance decreases when the lag distance exceeds 1000 m, which corresponds to the minimum distance between observations in our dataset.

**
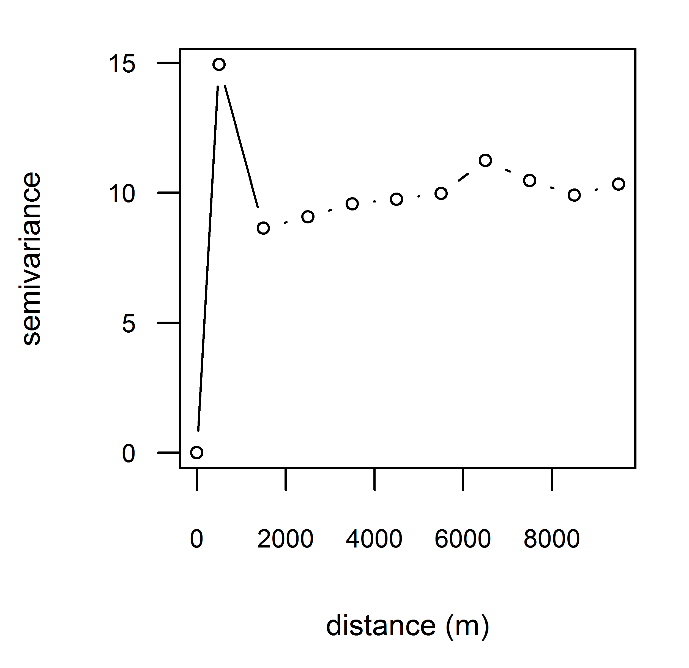
**

**Appendix 2.** Summary of regional and local scale models testing a) the importance of geology and terrain for rainforest density at a regional scale, and the importance of geology alone on b) complex terrain and c) level terrain; the effects of insularity (mainland vs islands) and terrain (complex vs level) at a local scale on d) rainforest density, and e) fire activity. *K* represents the number of parameters, *AIC* the Akaike value, *Delta* the difference between the AIC of the model and the AIC of the best model in the set, *Weight* the Akaike weight (defined as the relative likelihood of the model), and *Deviance* is the percentage of deviance explained by the model.

1. Rainforest density (Regional scale)

| Model | K | AIC | Delta | Weight | Deviance |
| --- | --- | --- | --- | --- | --- |
| Geology and terrain | 6 | 27898.0 | 0.00 | 1.00 | 32.0% |
| Terrain | 2 | 28949.6 | 596.31 | 0.00 | 26.3% |
| Geology | 5 | 28949.6 | 1051.52 | 0.00 | 19.4% |
| Null | 1 | 30133.7 | 2235.65 | 0.00 | NA |

1. Rainforest density on complex terrain (Regional scale)

| Model | K | AIC | Delta | Weight | Deviance |
| --- | --- | --- | --- | --- | --- |
| Geology | 5 | 13085.8 | 0.00 | 1.00 | 13.0% |
| Null | 1 | 13537.8 | 452.00 | 0.00 | NA |

1. Rainforest density on level terrain (Regional scale)

| Model | K | AIC | Delta | Weight | Deviance |
| --- | --- | --- | --- | --- | --- |
| Geology | 5 | 15164.8 | 0.00 | 1.00 | 10.0% |
| Null | 1 | 15427.9 | 263.10 | 0.00 | NA |

1. Rainforest density (Local scale)

| Model | K | AIC | Delta | Weight | Deviance |
| --- | --- | --- | --- | --- | --- |
| Insularity and terrain | 3 | 7001.4 | 0.00 | 1.00 | 35.0% |
| Insularity | 2 | 7278.2 | 276.82 | 0.00 | 21.7% |
| Terrain | 2 | 7451.1 | 449.75 | 0.00 | 12.6% |
| Null | 1 | 7669.5 | 668.15 | 0.00 | NA |

1. Fire activity (Local scale)

| Model | K | AIC | Delta | Weight | Deviance |
| --- | --- | --- | --- | --- | --- |
| Insularity | 2 | -1402.0 | 0.00 | 0.52 | 39.1% |
| Insularity and terrain | 3 | -1401.8 | 0.12 | 0.48 | 39.2% |
| Terrain | 2 | -581.7 | 820.26 | 0.00 | 0.4% |
| Null | 1 | -576.9 | 825.04 | 0.00 | NA |

**Appendix 3.** Accuracy of the north Kimberley rainforest map, assessed through aerial survey.

|  | | | Habitat | |
| --- | --- | --- | --- | --- |
|  |  |  | Rainforest | Savanna |
| Producer's accuracy (omission) | | |  |  |
| In class (%) | | | 83 | 95 |
| Not in class (%) | | | 17 | 5 |
| User's accuracy (commission) | | |  |  |
| In class (%) | | | 82 | 95 |
| Not in class (%) | | | 18 | 5 |
| Overall accuracy (%) | 93 |  |  |  |
| Kappa coefficient | 0.78 |  | |  |

**Appendix 4.** Average rainforest density, measured as ha · km^-2^, on complex and level terrain and on different geology in the north Kimberley.

| Geology | Rainforest density (ha · km^-2^) | |
| --- | --- | --- |
|  | Complex terrain | Level terrain |
| Alluvium and colluvium | 0.11 ± 0.11 | 0.05 ± 0.02 |
| Basalt | 5.56 ± 0.28 | 0.33 ± 0.05 |
| Coastal sediments | 6.21 ± 1.96 | 0.83 ± 0.50 |
| Laterite | 18.87 ± 2.32 | 1.22 ± 0.17 |
| Sandstone | 1.38 ± 0.11 | 0.16 ± 0.01 |
